# Artificial Intelligence in Non-invasive Hemodynamic Monitoring: A Systematic Review of Accuracy, Effectiveness, and Clinical Applicability in Cardiology

**DOI:** 10.7759/cureus.92792

**Published:** 2025-09-20

**Authors:** Anas E Ahmed, Suhail M Al-Kinani, Abdulrahman M Alshammari, Raghad F Alharbi, Ghadeer S Alaydaa, Reem M Alanazi, Murad A Sharif, Jod N Refaei, Hanan N Abu Summah, Daniyah H Altubayqi, Salman M Alhubail, Abdulbari M Bannan, Ahmed Y Hurubi

**Affiliations:** 1 Community Medicine, Jazan University, Jazan, SAU; 2 College of Medicine, Jazan University, Jazan, SAU; 3 College of Medicine, Hail University, Hail, SAU; 4 College of Medicine, King Abdulaziz University, Jeddah, SAU; 5 College of Medicine, Tabuk University, Tabuk, SAU; 6 College of Medicine, Ibn Sina National College, Jeddah, SAU; 7 College of Medicine, King Faisal University, Al-Ahsa, SAU; 8 Internal Medicine, King Fahad Central Hospital, Jazan, SAU

**Keywords:** artificial intelligence, cardiology, cardiovascular care, coronary artery disease, diagnostic accuracy, electrocardiography, hemodynamic monitoring, non-invasive monitoring, predictive analytics

## Abstract

Hemodynamic monitoring is essential in cardiology for guiding diagnosis and therapy, but conventional invasive methods carry procedural risks while non-invasive methods often lack accuracy. The integration of artificial intelligence (AI) into monitoring devices offers opportunities to improve predictive accuracy, diagnostic yield, and workflow efficiency. This systematic review evaluated the role of AI-enhanced non-invasive hemodynamic monitoring devices in cardiology, focusing on effectiveness, accuracy, and clinical applicability. A comprehensive search of PubMed, Scopus, Web of Science, and Cochrane CENTRAL from inception to November 2024 retrieved 4,856 records; after duplicate removal, 4,158 articles were screened, 23 full texts were assessed, and nine studies met the inclusion criteria. Across diverse populations and settings, AI models consistently outperformed conventional approaches in predicting circulatory failure and hypotension, with an area under the receiver operating characteristic curve exceeding 0.90 in several studies. Non-invasive diagnostic enhancements included AI-electrocardiography for coronary artery disease detection and automated coronary calcium scoring from computed tomography scans with near-perfect agreement to expert readers. Invasive imaging applications demonstrated faster and more accurate intravascular ultrasound analysis, while workflow-focused systems reduced alarm fatigue without compromising safety. Most studies were of good methodological quality, although limitations included retrospective designs, heterogeneous populations, and limited prospective validation. Overall, AI-enhanced non-invasive hemodynamic monitoring shows strong potential to shift cardiovascular care from reactive detection to predictive and proactive management by improving accuracy, efficiency, and usability across diagnostic and monitoring domains, but large-scale prospective trials are needed to confirm real-world clinical impact, ensure equitable adoption, and address challenges related to interpretability and integration.

## Introduction and background

Hemodynamic monitoring is central to cardiovascular care, offering critical insights into blood pressure, cardiac output, vascular resistance, and circulatory status that guide therapy in intensive care, perioperative, and outpatient settings [1]. Invasive methods such as pulmonary artery catheterization and arterial line placement remain the gold standard for precision but carry risks of infection, vascular injury, and thrombosis, restricting their use to controlled environments [2]. To overcome these limitations, non-invasive approaches including electrocardiography (ECG), photoplethysmography (PPG), impedance cardiography, and tonometry have been developed. These technologies are safer and more feasible in ambulatory settings but often sacrifice accuracy, particularly in unstable patients [3].

The integration of artificial intelligence (AI), machine learning (ML), and deep learning (DL) into cardiology offers opportunities to enhance non-invasive monitoring by analyzing complex physiological data, identifying subtle patterns, and predicting outcomes with greater precision than conventional techniques [4]. AI has already shown success in imaging interpretation, arrhythmia detection, and outcome prediction, and its application to hemodynamic monitoring may transform devices into adaptive systems for early risk stratification and personalized care [5].

Recent studies highlight this potential. AI applied to ECG and wearable monitors has accurately detected coronary artery disease, predicted elevated left atrial pressures, and identified impending hemodynamic deterioration [6]. AI-driven analysis of computed tomography has achieved near-perfect agreement with experts in coronary calcium scoring [7]. ML models applied to arterial waveforms have predicted hypotension minutes before onset, enabling proactive intervention [8]. These advances suggest that AI-enhanced non-invasive monitoring could combine the safety of non-invasive tools with accuracy approaching invasive methods.

However, research remains fragmented across diagnostic, predictive, and workflow-focused applications, with heterogeneous populations and outcomes limiting comparability [9]. No prior systematic review has comprehensively synthesized evidence on AI-enhanced non-invasive hemodynamic monitoring in cardiology. This review therefore evaluates their accuracy, reliability, and clinical impact, aiming to clarify current evidence, highlight benefits and limitations, and identify future research directions.

## Review

Methods

Literature Search Strategy

This review followed the Preferred Reporting Items for Systematic Reviews and Meta-Analyses (PRISMA) guidelines [10]. A comprehensive search was conducted in PubMed, Scopus, Web of Science, and Cochrane CENTRAL from inception to July 10, 2025, using controlled vocabulary and free-text terms: (“Artificial Intelligence” OR “Machine Learning” OR “Deep Learning”) AND (“Hemodynamic Monitoring” OR “Cardiac Output” OR “Blood Pressure” OR “Non-invasive”) AND (“Cardiology” OR “Heart Disease”). Search strategies were adapted for each database, focusing on English-language studies that involved human participants.

Eligibility Criteria

Eligibility was defined using the Population, Intervention, Comparator, Outcomes, Study design (PICOS) framework [11]. Included studies were original research involving human participants in cardiac care, perioperative management, or intensive care, applying AI, ML, or DL to non-invasive or minimally invasive hemodynamic monitoring tools (e.g., ECG, wearable sensors, PPG, arterial waveform analysis). Studies had to compare AI-enhanced methods against conventional reference standards such as coronary angiography, echocardiography, or expert interpretation, reporting outcomes on diagnostic accuracy, predictive validity, clinical utility, or workflow efficiency. Reviews, case reports, conference abstracts without full text, non-human studies, and studies lacking a clear AI component were excluded.

Study Selection

Two reviewers independently screened titles and abstracts, retrieving full texts for potentially eligible studies. Discrepancies were resolved through discussion with a third reviewer until consensus was reached.

Data Extraction

Key information was extracted from included studies, including study identification, country, design, population, setting, device or technology, AI methodology, comparators, outcomes, and main results. Extraction was performed independently by two reviewers, with inconsistencies resolved by a third reviewer.

Quality Appraisal

Methodological quality was assessed using the modified 28-item Downs and Black checklist [12], evaluating reporting, external validity, internal validity, and power. Scores ranged from 0 to 28 and were classified as excellent (26-28), good (20-25), fair (15-19), or poor (≤14). Disagreements were resolved through discussion until a consensus was reached.

Results

Study Selection

**Figure 1:**
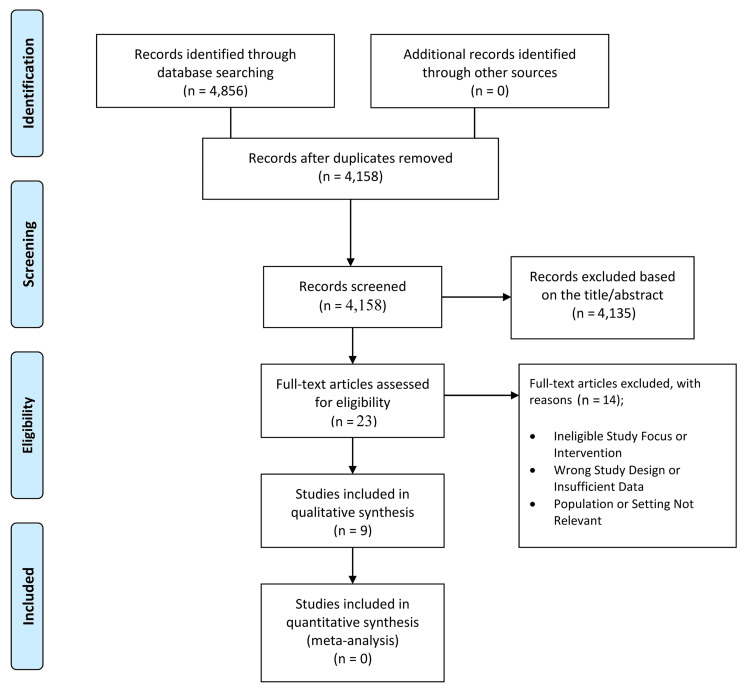
PRISMA flow diagram of the study selection process PRISMA: Preferred Reporting Items for Systematic Reviews and Meta-Analyses

A total of 4,856 records were identified through database searches. After removing duplicates, 4,158 records were screened, excluding 4,135 for irrelevance or ineligibility. Twenty-three full-text articles were assessed, with 14 excluded for ineligible focus, design, or population. Nine studies met all criteria and were included in the qualitative synthesis, as shown in the PRISMA flow diagram (Figure 1).

Study Characteristics

The studies highlight diverse AI applications in cardiology, from diagnostic enhancements to predictive monitoring (Table 1). AI applied to 12-lead ECGs achieved area under the curve (AUC) values up to 0.91 for coronary artery disease [13]. Convolutional neural networks automated coronary calcium scoring from CT scans with near-perfect agreement with expert readers and substantially faster processing [14].

**Table 1:** AI-enhanced non-invasive and minimally invasive hemodynamic monitoring studies in cardiology This table summarizes key characteristics, AI/machine learning methods, comparators, outcomes, and main results of included studies evaluating AI-enhanced non-invasive and minimally invasive hemodynamic monitoring technologies in cardiology. AI, Artificial Intelligence; ML, Machine Learning; ECG, Electrocardiography; CAD, Coronary Artery Disease; CT, Computed Tomography; CAC, Coronary Artery Calcium; NIRS-IVUS, Near-Infrared Spectroscopy–Intravascular Ultrasound; DL, Deep Learning; Bi-GRU, Bidirectional Gated Recurrent Unit; ICU, Intensive Care Unit; MAP, Mean Arterial Pressure; AUC / AUROC, Area Under the (Receiver Operating Characteristic) Curve; AUPRC, Area Under the Precision-Recall Curve; HPI, Hypotension Prediction Index; PCI, Percutaneous Coronary Intervention; IVUS, Intravascular Ultrasound; TWA MAP, Time-Weighted Average Mean Arterial Pressure; RHC, Right Heart Catheterization; LAP, Left Atrial Pressure; mPCWP, Mean Pulmonary Capillary Wedge Pressure; HR, Heart Rate; RR, Respiratory Rate; SpO₂, Peripheral Oxygen Saturation

Study ID	Country	Study Design & Population	Setting	Device/Technology Characteristics	AI / Machine Learning Component	Comparators	Outcomes	Results Summary
Yeh et al. [13]	Taiwan	Retrospective study; Training cohort n=5,376 and Validation cohort n=7,163; adults ≥18 years undergoing coronary angiography	Chang Gung Memorial Hospital (Keelung & Linkou branches)	12-lead resting ECG; dataset of 239,474 ECGs (final analyzed ~12,500)	XGBoost model; 561 ECG-derived features (intervals, amplitudes, slopes); SHAP framework for feature importance	Coronary angiography (gold standard); myocardial perfusion scintigraphy; conventional ECG	Sensitivity 0.82–0.84; AUC up to 0.91; accuracy 0.85; comparable to Thallium scan	AI-enhanced ECG improved CAD detection, including in normal ECGs; identified novel ECG features (slope, amplitude differences); showed sex-specific patterns; offers a non-invasive alternative with nuclear imaging–level accuracy
Ihdayhid et al. [14]	Australia, UK, Canada	Retrospective, multicenter observational study; Training set: 2,439 cardiac CT scans; Validation: 771 scans; Independent test: 1,849 patients (mean age 55.7 ± 10.5 years, 49% male)	Fiona Stanley Hospital (Australia), University of Edinburgh (UK), Envision Medical Imaging (Australia), University of Ottawa Heart Institute (Canada)	Non-contrast ECG-gated cardiac CT scans; Agatston CAC scoring	3D fully convolutional neural network (DeepC architecture) with additional CNNs for aortic and cardiac segmentation; trained on Siemens, Philips, Toshiba CT datasets	Manual CAC scoring by level 3 expert readers	Strong correlation with manual scoring (Spearman’s r = 0.90; ICC = 0.98); 89% agreement in risk category classification (κ = 0.90); mean analysis time 13.1 ± 3.2 s/scan	AI model detected CAC with high accuracy, low analysis time, and strong agreement with manual scoring; reduced workload potential; slight misclassification at very low CAC levels; model not yet validated in noisy/artefact scans.
Hyland et al. [15]	Switzerland (development), external validation in the USA	Retrospective cohort; 36,098 ICU admissions, >45,000 circulatory failure events	Bern University Hospital ICU; external validation in Beth Israel Deaconess ICU (MIMIC-III)	High-resolution ICU electronic patient data management system (HiRID dataset, 7,333 variables, 3B observations, sampled every 2 min)	Gradient-boosted decision trees (LightGBM); circEWS (500 features) and circEWS-lite (176 features); SHAP for interpretability	Baseline decision-tree model using MAP, lactate, vasopressors; conventional threshold-based alarms	AUROC 0.94 (full), 0.939 (compact) vs 0.883 baseline; AUPRC 0.467/0.454 vs 0.254 baseline; 82% events predicted >2 h in advance; alarm rate 0.05 per patient-hour	ML-based early-warning system accurately predicted circulatory failure up to 8 h in advance with fewer false alarms; externally validated; minor limitations in neurological patients; demonstrates clinical feasibility
Hatib et al. [16]	USA & international	Retrospective cohort; training 1,334 patients, internal 350, external 204	Operating rooms and ICUs (multi-site)	High-fidelity invasive arterial pressure waveforms (100–500 Hz), FloTrac/CO-Trek algorithms	Logistic regression ML algorithm using 3,022 waveform-derived features; output = Hypotension Prediction Index (HPI)	Standard MAP thresholds (<65 mmHg) and ΔMAP changes	Internal: AUC 0.95, sensitivity 87.5%, specificity 87.3%; External: AUC 0.91, sensitivity 83.6%, specificity 83.3%; superior to ΔMAP	HPI predicted hypotension up to 15 min in advance with high accuracy; outperformed standard metrics; potential to reduce intraoperative complications; limitations include reliance on invasive arterial lines
Kouz et al. [17]	Multicenter (France, Germany, Italy, Spain, UK)	Prospective multicenter observational registry; 702 patients undergoing elective major noncardiac surgery under GA	12 European medical centers	Acumen™ IQ sensor + HemoSphere platform with HPI software	ML-based HPI algorithm analyzing arterial pressure waveform features; alarm threshold HPI >85	No randomized comparator; descriptive analysis	Median TWA MAP <65 mmHg = 0.03; median duration <65 mmHg = 2 min; 3% myocardial injury; 9–11% acute kidney injury; 2% 30-day mortality	Registry patients experienced very low exposure to intraoperative hypotension, which suggests HPI monitoring may reduce severity/duration; limitations: observational, no direct comparator, outcomes exploratory.
Schlesinger et al. [18]	USA	Retrospective development: 6,739 samples; external validation: 4,620 samples; prospective patch-monitor cohort: 83 patients undergoing RHC	Hospital catheterization labs; step-down unit	Wearable patch ECG monitor recording single-lead ECG (256 Hz)	Deep residual neural network (CHAIS); pre-trained,t hen fine-tuned for mPCWP >18 mmHg classification; entropy-based trustworthiness score	Right heart catheterization (mPCWP ≥18 mmHg)	AUROC 0.80–0.875; sensitivity 70–80%, specificity 66–82%; NPV >95% at low prevalence	CHAIS reliably predicted elevated LAP/mPCWP from single-lead ECG; performance comparable to echo Doppler; feasible for ambulatory HF monitoring; limitations: small prospective cohort, generalizability uncertain
Min et al. [19]	South Korea	Retrospective cohort; 618 patients with 618 coronary lesions undergoing PCI; 34,736 IVUS frames	Asan Medical Center	IVUS with pre- and post-stenting co-registration; drug-eluting stents	Deep learning models: DenseNet CNN for stent area, FCN-VGG16 for IVUS segmentation, XGBoost for underexpansion	Post-stenting IVUS measurements (minimal stent area <5.5 mm² = underexpansion)	Frame-level: AUC 0.94, accuracy 94%; Lesion-level: correlation r=0.832–0.958; overall accuracy 93%	DL algorithms accurately predicted stent underexpansion using pre-procedural IV, S, enabling preemptive PCI planning; limitations: retrospective, single-center, no long-term outcome validation
Bajaj et al. [20]	UK (Barts Heart Centre, London)	Prospective study; 20 coronary arteries from six patients with stable angina undergoing NIRS-IVUS imaging; 92,526 frames analyzed	Barts Heart Centre, Queen Mary University of London	NIRS-IVUS at 30 fps and 15 fps; ECG co-registered	Deep learning model using a Bi-GRU network; trained on frame sequences (64 frames) with vessel-specific training (LAD, LCx, RCA)	ECG-defined end-diastolic frames; expert analysts; conventional image-based methodology	Accuracy of DL = 80.4% vs analysts (39–43%) and conventional image-based (42.8%); RMSE significantly lower for DL	DL methodology outperformed experts and conventional methods in accuracy and reproducibility, rapid processing, robust across vessels, and potential for integration into clinical IVUS software.
Ruppel et al. [21]	USA	Pre/post intervention study; patients: N=677 pre, N=659 post; nurses: 66 pre, 44 post	Yale New Haven Hospital Medical ICU	IntelliVue® Alarm Advisor software integrated with monitors; visual alerts for repeated/prolonged medium-priority alarms	Customization support algorithm (rule-based AI-driven alarm management)	Pre- vs post-implementation monitoring of alarms; nurse surveys	Medium-priority HR, RR, and arterial pressure alarms reduced 9–16%; duration reduced 7–13%; nurses reported lower disturbance	Alarm customization reduced alarm burden and improved nurse experience without increasing workload; limitations: single-center, pre/post design, low survey response rate, no direct measure of alarm validity

Predictive monitoring studies demonstrated early detection of hemodynamic instability. Gradient-boosted models predicted circulatory failure hours in advance with an area under the receiver operating characteristic curve (AUROC) of 0.939-0.940 [15]. The Hypotension Prediction Index accurately anticipated intraoperative hypotension (AUC>0.9) and reduced hypotensive episodes in multicenter registries [16,17]. Wearable ECG patches with DL predicted elevated pulmonary capillary wedge pressure with AUROCs up to 0.875 and negative predictive values over 95% [18].

Other studies explored procedural and workflow improvements. DL enhanced intravascular ultrasound (IVUS) analysis, predicting stent under-expansion with frame-level accuracies of 94% [19] and automating end-diastolic frame detection, halving analysis time [20]. AI-driven alarm customization reduced non-actionable ICU alarms by 9-16% without increasing adverse events, improving workflow efficiency [21].

Quality Assessment

Using the Modified Downs and Black checklist, most studies were rated “good.” Reporting quality was high, while external validity varied, with multicenter studies scoring higher than single-center ones [14,17]. Internal validity was generally strong, though limitations included blinding, selection, and confounder adjustment. Overall scores ranged from 17 to 23/28, highlighting robust evidence in larger studies but higher bias risk in smaller studies [15,17,18,20,21] (Table 2).

**Table 2:** Methodological quality assessment of included studies using the modified Downs and Black checklist This table summarizes the methodological quality of included studies based on the modified Downs and Black checklist. Scores are provided for each domain: Reporting (clarity of objectives, methods, and outcomes; 0–10), External Validity (generalizability; 0–3), Internal Validity – Bias (risk of bias; 0–7), Internal Validity – Confounding (control for confounders; 0–6), and Power (sample size justification; 0–1). Total scores range from 0 to 28, with higher scores indicating better methodological quality.

Study ID	Reporting (0–10)	External Validity (0–3)	Internal Validity – Bias (0–7)	Internal Validity – Confounding (0–6)	Power (0–1)	Total (0–28)
Yeh et al. [13]	9	2	5	4	0	20
Ihdayhid et al. [14]	9	3	6	4	0	22
Hyland et al. [15]	9	2	6	4	0	21
Hatib et al. [16]	9	2	6	4	0	21
Kouz et al. [17]	9	3	6	4	1	23
Schlesinger et al. [18]	9	2	6	4	0	21
Bajaj et al. [20]	8	1	5	3	0	17
Ruppel et al. [21]	8	1	5	3	0	17

Effect of Prediction of Circulatory Failure and Hypotension

AI models consistently predicted hemodynamic deterioration before onset. Gradient-boosted ensembles predicted circulatory failure up to eight hours in advance (AUC 0.939-0.940), with good calibration across patient groups [15]. The Hypotension Prediction Index forecasted intraoperative hypotension up to 15 minutes ahead (AUC>0.9) [16], with registry data showing reduced hypotensive episodes and low postoperative complications [17]. Wearable ECG-based models predicted elevated left atrial pressures with AUROCs of 0.76-0.875 and high negative predictive values [18].

Effect of AI-Enhanced Diagnostic Use of Non-invasive Tests

AI improved the diagnostic yield of routine tests. ECG-based models detected coronary artery disease with an AUC of 0.91, identifying disease even in patients with normal conventional ECGs [13]. AI automated coronary calcium scoring from CT scans, achieving near-perfect agreement with experts and reducing analysis time to seconds [14].

Effect of AI for Invasive Imaging Optimization

Deep learning applied to IVUS predicted stent underexpansion with frame-level AUC 0.94 [19]. Automated end-diastolic frame detection improved accuracy and reduced analysis time from 15 minutes to 15 seconds [20], enhancing efficiency and reproducibility in invasive imaging.

Effect of AI for Alarm and Workflow Optimization

AI-driven alarm management reduced medium-priority ICU alarms by 9-16% and improved nurse experience without increasing adverse events [21], showing potential to enhance workflow and safety in hemodynamic monitoring.

Discussion

The findings of this systematic review underscore the growing impact of AI-enhanced non-invasive and minimally invasive technologies in cardiology. Across the included studies, AI consistently outperformed conventional methods in predicting circulatory failure and hypotension, improving the diagnostic yield of routine tests such as ECG and CT, optimizing invasive imaging, and reducing alarm burden in clinical monitoring. Collectively, these results suggest that AI can shift hemodynamic monitoring from a reactive, threshold-based approach to a predictive, adaptive, and efficient paradigm, potentially improving patient outcomes and workflow efficiency. Predictive models demonstrated strong performance in perioperative and ICU settings, with AI-enabled systems accurately forecasting hypotension and elevated filling pressures even in ambulatory patients. The integration of alarm mitigation strategies further highlights AI’s ability to enhance both patient safety and clinician usability, supporting a more proactive and manageable monitoring environment.

Beyond predictive monitoring, AI also shows promise in diagnostic and procedural applications. Enhanced ECG and automated coronary calcium scoring achieved accuracy comparable to expert interpretation while substantially reducing analysis time, suggesting that AI can expand access to early and precise cardiovascular disease detection. Invasive imaging workflows, such as IVUS, benefited from AI-driven frame selection and stent expansion prediction, improving efficiency and reproducibility. Additionally, AI-driven alarm customization and multi-parameter monitoring addressed workflow challenges, reducing non-actionable alerts and cognitive burden on clinical staff. Taken together, these findings indicate that AI has the potential to integrate diverse data streams-non-invasive or invasive-into actionable clinical insights, enhancing diagnostic precision, proactive patient management, and overall care efficiency in cardiovascular practice.

Several limitations should be considered. Most included studies were retrospective or observational, with few prospective, multicenter designs, which limits the generalizability of findings. The heterogeneity of populations, devices, and outcomes also precluded direct quantitative synthesis. Many studies excluded complex cases, noisy data, or patients with multiple comorbidities, potentially leading to overestimation of algorithm performance compared with real-world practice. Additional challenges include the “black box” nature of AI models, difficulties integrating them with electronic health records, and concerns regarding data privacy and bias, all of which remain barriers to clinical translation.

## Conclusions

This systematic review shows that AI-enhanced non-invasive hemodynamic monitoring holds substantial potential to transform cardiovascular care by improving diagnostic accuracy, enabling predictive monitoring, and streamlining workflows. While current evidence is promising, large-scale, and prospective, diverse clinical trials are needed to validate these systems in real-world settings, address interpretability and equity concerns, and ensure seamless integration into clinical practice. As the technology advances, AI should be viewed not as a replacement for clinical expertise but as a complementary tool that empowers clinicians to deliver safer, more personalized, and proactive cardiovascular care.
